# Industrial Fatigue

**Published:** 1916-04-08

**Authors:** 


### The Hospital

The Workers' Newspaper of Administrative Medicine and Institutional Life, Administration, National Insurance and Health.

No. 1556, Vol. LX. SATURDAY, APRIL 8, 1916. PRICE ONE PENNY

### INDUSTRIAL FATIGUE.

Next to the application of science to warfare the application of science to industry seems to be the question of the hour. Our universities and colleges are being told that their programmes are not sufficiently practical, and our manufacturers and traders learn that they rely too much on rule of thumb. In both of these respects it is plainly intimated there must be improvement if the nation is to hold its own in the industrial war which is to follow the proclamation of European peace. Probably most readers assent to these propositions under the belief that they apply to investigations and methods and processes and materials, as without question they certainly do. Chemical knowledge and the devising and use of machines also probably receive recognition. But not everyone will have thought of the need for the scientific study of the worker. Yet here is a machine which is employed on the widest possible scale, and on the efficiency of which all other activities—manufacturing and manual not less than inventive and mental—most certainly depend. If science is to be applied to industry one part of the application must relate to the physiological units by which the industry is accomplished. The methods and materials of course, but also, and not less, the man.

This general truth has been recognised by the Committee charged with the duty of investigating the health of the workers in war munitions, and the recently issued report of the Committee is full of interest and of practical suggestion. We refer here more particularly to the section of the report which deals with industrial fatigue and its causes, and we may say at once that the substance of this section is of high importance in relation both to the present emergency and to the future industrial efficiency of the nation. Put briefly and in general terms, the conclusion announced may be defined as a plea for organised and scientifically determined rest as a necessary condition not only for the welfare of the individual worker but also for the highest quantitative and qualitative output. The natural exhortation of the practical man is for longer hours, and more overtime, and less leisure, if the demand for larger results is to be met. But in the view of the Committee this exhortation has in the present crisis already been pushed too far, and under it the munition workers in the country generally have been allowed to reach a state of reduced vigour and lowered health which might have been avoided without prejudice to the total output by more attention to the details of the daily and weekly rests. In all this it will be observed the Committee is concerned not with what may be called a sentimental or humanitarian point of view, but strictly with practical results as these are measured by output in its quantitative and qualitative relations. Other considerations apart, it is grateful to find that the two points of view are not in conflict the one with the other, and that to secure the success of the nation it is not necessary to demand the sacrifice of the individual. Both the one aim and the other are to be attained by one and the same influence.

The strictly practical part of the Committee's report is prefaced by an exposition of physiological doctrine which is well worthy of careful study and is in itself a strong argument for scientific tests as opposed to mere empirical determinations. Tested by these latter, fatigue would be recognised as revealing itself by certain familiar bodily sensations. These are generally regarded as announcing its arrival and measuring its degree, and they are in practice referred to the muscular system. It is, however, certain that in all ordinary circumstances there is never produced anything like advanced fatigue of the muscles. Given a new or more powerful stimulus, these, even in a "tired" man, still display a large capacity for work. What really happens is that the nervous machinery suffers fatigue long prior to the muscular, and the sensation of tiredness is an announcement of this fact. Still more important is the observation that while this sensation of tiredness is the first coarse index of the arrival of fatigue, its absence is not a guarantee that the capacity for work has not yet been diminished and that so far rest is not needed. On the contrary, it is certain that if measurement is determined by this standard and if the periods of work and rest respectively are adjusted by it, many erroneous conclusions will be formed and many unfruitful practices will be arranged. In a word, bodily sensations are not a true guide as to the existence of fatigue in the sense that by this condition is meant a diminished capacity for doing work. When this diminution arrives, however it may be determined, there is a physiological need for rest, so that once again the bodily machinery may be raised to a high degree of efficiency. Granted these two propositions and it becomes obvious that the organisation of industrial labour is a physiological problem which is to be solved by the determination of a scientific standard and by accurate observations and accurate measurements.

Fortunately it seems clear that these ends can be gained by very direct methods and independently of any procedures which might smell too strongly of the laboratory. As the true sign of fatigue is diminished capacity it follows that measurement of output in work will give the most direct test of fatigue. But to be of value these measurements must be made not in large figures or mere end results. They must be estimated under the conditions which are attached to any other scientific problem; and apparent exceptions to what seems to be a general rule must receive particular attention. Thus, as an example of this last-mentioned demand, it has been noted that an output above the average made by some given individual is often the result of escape from fatigue by conscious or unconscious adoption of particular habits of manipulation or of rhythm. From such a discovery flows an opportunity of instruction for all other workers. There could be no more forcible illustration of the value of the kind of study of the human machine which the Committee recommends. The evidence is plain that the absence of an adequate measure of rest means not only lessened output but also a greater number of accidents, more spoiled work, and an increased toll of sickness. Thus on every hand there is reason to urge that the hours of the workers and the adjustment of resting periods ought to be determined not by some chance or arbitrary standard but by physiological doctrine, as this is decided by carefully collected observations.

